# Epigenetics in Alzheimer’s Disease

**DOI:** 10.3389/fnagi.2022.911635

**Published:** 2022-06-23

**Authors:** Xiaodie Gao, Qiang Chen, Hua Yao, Jie Tan, Zheng Liu, Yan Zhou, Zhenyou Zou

**Affiliations:** ^1^Guangxi Key Lab of Brain and Cognitive Neuroscience, Guilin Medical University, Guilin, China; ^2^Department of Scientific Research, Brain Hospital of Guangxi Zhuang Autonomous Region, Liuzhou, China

**Keywords:** Alzheimer’s disease, epigenetics, methylation, acetylation, ubiquitination

## Abstract

Alzheimer’s disease (AD) is a neurodegenerative disease with unknown pathogenesis and complex pathological manifestations. At present, a large number of studies on targeted drugs for the typical pathological phenomenon of AD (Aβ) have ended in failure. Although there are some drugs on the market that indirectly act on AD, their efficacy is very low and the side effects are substantial, so there is an urgent need to develop a new strategy for the treatment of AD. An increasing number of studies have confirmed epigenetic changes in AD. Although it is not clear whether these epigenetic changes are the cause or result of AD, they provide a new avenue of treatment for medical researchers worldwide. This article summarizes various epigenetic changes in AD, including DNA methylation, histone modification and miRNA, and concludes that epigenetics has great potential as a new target for the treatment of AD.

## Introduction

Alzheimer’s disease (AD) is a progressive neurodegenerative disease with an incidence of 10% in people over 65 years old and 40% in people over 85 years old, and the number of confirmed cases is expected to increase to over 91 million worldwide by 2050 (WHO, 2017); it comprises more than 2/3 of confirmed cases of dementia (Robinson et al., 2018). A loss of short-term memory, reduced sense of direction, decreased expression ability and progressive changes in personality are typical clinical manifestations of AD (Panza et al., 2019). AD can be classified into two main types: early-onset AD (EOAD) and late-onset AD (LOAD). EOAD develops cognitive impairment before the age of 65 and accounts for approximately 5% of all cases; LOAD develops cognitive impairment after the age of 65 and accounts for more than 90% of diagnosed AD patients (Diniz et al., 2017).

As confirmed by a large number of studies, the main pathological manifestations of AD are extraneuronal amyloid plaque (Aβ) deposition and intracellular neurofibrillary tangles (NFTs) (Krance Saffire et al., 2019; No authors listed, 2021). Hyperphosphorylated tau protein, which has over 40 phosphorylation sites, is the main component of the tangles (No authors listed, 2021). A recent autopsy study indicated that p-tau at threonine 217 (p-tau217) was the most important phosphorylation site in the differentiation between Alzheimer’s disease and control brain tissue (Wesseling et al., 2020), and plasma p-tau217 has been considered a biomarker of AD (Thijssen et al., 2021). Aβ and tau protein deposition can affect signal and substance transmission between neurons, leading to neuronal degeneration and death (Bottero et al., 2021).

**FIGURE 1:**
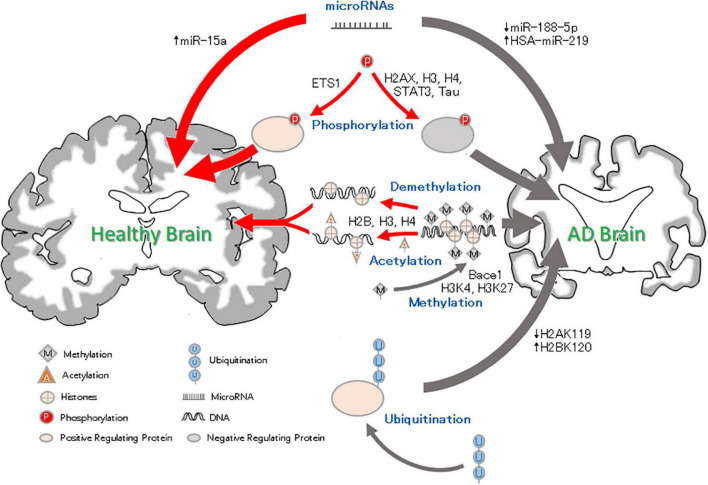
Epigenetic factor roles in Alzheimer’s disease. Methylation and Acetylation: DNA methylation results in reduced silencing of neurodegenerative genes and affects neural development. Histone methylation may condense chromosomes, thus preventing the expression of binding genes. Histone acetylation leads to chromatin becoming more open, which promotes gene expression. Phosphorylation: Histone H2AXS139 and H3 and H4S473 phosphorylation can lead to neurodegeneration. Ets1 phosphorylation reverse the pathological AD changes. Ubiquitation: High ubiquitination of H2BK120 and low ubiquitination of H2A119 and Trk1 can be related to the pathology of AD and aggravate the AD process. MicroRNA: microRNAs can inhibit gene expression. The downregulation of miRNA-188-5p and upregulation of HAS-miRNA-219 can lead to cognitive impairment, but an increase in miRNA-15a can inhibit neuronal apoptosis and alleviate AD.

During the preclinical stage, subjects are cognitively unimpaired but show evidence of cortical Aβ deposition, which is considered the most upstream process in the pathological cascade of Alzheimer’s disease (Jack et al., 2013). There are reports that in AD familial mutation carriers, Aβ starts accumulating over two decades before any symptoms appear, followed by brain metabolism decline 6 years prior to and brain atrophy approximately 5 years prior to any symptoms (Gordon et al., 2018). In view of the hypothesis that Aβ accumulation is the underlying etiology, researchers have conducted a large number of in-depth and extensive studies aimed at revealing the pathogenesis of AD to find ways to treat AD. Unfortunately, these studies all ended in failure. In the face of the global surge in the diagnosed population of AD, it is urgent to find a new direction of treatment for AD, shown in Figure 1.

In the middle of the twentieth century, Waddington first linked developmental biology with genetics and put forward the concept of “epigenetics” (Holliday, 2006). With the continuous progress of research, the concept of epigenetics is constantly improving. At present, epigenetics is generally defined as “to make structural and biochemical changes in chromatin without changing the DNA sequence, and then regulate the expression of related genes, thus affecting various physiological and pathological processes” (Wang et al., 2019; Li, 2021). These changes include DNA methylation and hydroxymethylation, histone modifications (histone methylation, acetylation, glycosylation, ubiquitination, phosphorylation), and non-coding RNA changes. Epigenetics has been shown to control the transcription of genes related to cell differentiation (Cantone and Fisher, 2013), learning and memory (Kosik et al., 2012) and has emerged as an important regulator of development and aging (Brunet and Berger, 2014; Orozco-Solis and Sassone-Corsi, 2014; Booth and Brunet, 2016; Pal and Tyler, 2016; Roberts et al., 2021), which is the biggest risk factor for AD.

Some studies have proposed that AD is not simply an advanced or exacerbated state of normal aging, but is instead a dysregulation of normal aging and normal aging might provide protection against AD-epigenetics may be involved (Fyfe, 2018). The occurrence and development of AD follow a non-Mendelian etiology, with both genetic and environmental modification factors (Zhang et al., 2019). Individuals carrying autosomal dominant Alzheimer’s disease mutations with near 100% penetrance develop dementia when aged approximately 30–60 years (Ryman et al., 2014; Fagan et al., 2021). However, not all ε4 carriers who survive to an advanced age develop AD, and an epigenomic factor associated with a reduced proportion of activated microglia (microglial epigenomic factor 1) appears to attenuate the risk of ε4 for AD (Ma et al., 2020).

In the AD brain, at the sub/cellular level, the dysregulation of several molecular pathways and intracellular signaling occurs, including Aβ and tau proteostasis, synaptic plasticity and homeostasis, inflammatory-immune responses, lipid and bioenergetic metabolism, and oxidative stress (Hampel et al., 2021), and their dysregulation results from a multilayer interaction among genetic, biological, and environmental factors (Castrillo and Oliver, 2016). In addition, increasing evidence has shown that an imbalance in epigenetic mechanisms may be the basis of abnormal expression of synaptic plasticity- and memory-related genes in AD (Mehler and Mattick, 2007; Vecsey et al., 2007; Guan et al., 2009; Michán et al., 2010). Here, we provide a brief review of the epigenetic changes in AD and further corroborate that epigenetic factors may be useful biomarkers to diagnose AD and therapeutic targets to treat AD.

## DNA Methylation and Hydroxymethylation in Alzheimer’s Disease

DNA methylation refers to the process of forming 5-methylcytosine (5mC) by a covalent bond with the cytosine 5′ carbon site of the CpG dinucleotide in the genome under the action of DNA methyltransferases (DNMTs) (Figure 1). 5mC can interfere with the binding of transcription factors to recognition sites on promoters or recruit transcription suppressor methyl-CpG-binding proteins to alter the chromatin structure and inhibit transcription (Klose and Bird, 2006; Kemme et al., 2017), thereby affecting gene expression (Jones and Laird, 1999; Cacabelos and Torrellas, 2014). 5mC can be oxidized into 5-hydroxymethylcytosine (5hmC), 5-formylcytosine (5fC) and 5-carboxylcytosine (5caC) by ten-eleven translocation (TET) family enzymes (Kudo et al., 2012; Fetahu et al., 2019). 5hmC is the intermediate product of DNA methylation and demethylation, which adds a layer of complexity to the epigenetic regulation of both. 5hmC has been found in various mammals (Tahiliani et al., 2009; Ito et al., 2010), especially in pluripotent stem cells and nerve cells with the ability to self-renew (Szwagierczak et al., 2010; Guo et al., 2011).

In contrast to 5mC, 5hmC is related to gene expression activation in the brain (Chen et al., 2012; Mellén et al., 2012; Colquitt et al., 2013). For example, the content of 5hmC in the brain of AD is positively correlated with the level of triggering receptor expressed on myeloid cells 2 (TREM2) gene (Celarain et al., 2016), which is supposed to promote the phagocytosis of Aβ_42_ peptide, preventing Aβ aggregation and downstream neurotoxic effects (Jiang et al., 2014; Zhao et al., 2014), especially in the hippocampus. In addition, 5hmC is also involved in physiological processes, such as cell differentiation, neural development and aging (Szulwach et al., 2011; Chouliaras et al., 2012; Wang et al., 2012). Some studies have found that 5hmC is selectively lost in hippocampal neurons and neocortical neurons in patients with AD and model mice, and this loss is significantly related to age and is aggravated after Aβ treatment. However, this change is not obvious in cerebellar neurons (Zhang et al., 2020). In addition, the loss of TET enzymes is consistent with that of 5hmC in the prefrontal cortex and hippocampus, and TET can inhibit the neuropathophenotype (Aβ aggregation, tau hyperphosphorylation) and prevent synaptic dysfunction in mice (Zhang et al., 2020). Moreover, the decrease in 5hmC and TET levels in the hippocampus of AD mice coincides with abundant Aβ plaque accumulation. In the early disease stage, the decline in TET levels in the hippocampus leads to a decrease in 5hmC content, which causes the appearance of pathological features, and these changes are alleviated after the restoration of TET expression (Li L. et al., 2020). Taken together, these results suggest that the loss of 5hmC, which is the result of the downregulation of TET, is closely related to the degree of AD neurodegeneration. However, the current research on 5hmC is not mature, and conflicting results have been reported in several studies (Chouliaras et al., 2013; Condliffe et al., 2014; Coppieters et al., 2014). The specific role of dynamic changes in 5hmC contributing to AD remains to be further explored.

5mC is the most well-studied epigenetic modification and it plays a critical role in brain development (Price et al., 2019; Monti et al., 2020; Meth et al., 2021). Studies have shown that the levels of 5mC in the middle temporal gyrus and middle frontal gyrus of AD patients increase significantly and are positively correlated with Aβ, NFTs and ubiquitin load (Coppieters et al., 2014). The entorhinal cortex is a critical brain region affected by Alzheimer’s disease (Howett et al., 2019). Researchers have found hypermethylation of the *ANK1* gene in the entorhinal cortex and similar methylation in the superior temporal gyrus and prefrontal cortex (De Jager et al., 2014; Wood, 2014). In addition, methylation changes have also been found in *ABCA7, BIN1* and other genes associated with the development of AD (De Jager et al., 2014; Wood, 2014). The hypermethylation of these genes is often accompanied by the deposition of large amounts of amyloid protein, suggesting that these changes are related to the pathology of AD, but these changes have also been found in the brains of some patients without cognitive impairment. In these patients, only low amounts of amyloid protein deposition were found, indicating that DNA methylation may be involved in the very early pathological changes of AD (De Jager et al., 2014). At present, only a small number of genetic variations described as risk factors for AD, such as genome-wide loci (IQCK, ACE, ADAM10, ADAMTS1, and WWOX), which include *ANK1*, *ABCA7, BIN1*, and others, are associated with LOAD (Kunkle et al., 2019; Sims et al., 2020).

In the 1990s, the pathogenic relationship between amyloid precursor protein (APP), presenilin1 (*PSEN1*) and AD was proven by genetic methodology, in which *PSEN1*, as the active site of the secretase, can influence the function of neuron γ secretase in AD patients, increase the level of plasma Aβ42 and accelerate the development of AD (Borchelt et al., 1996). The *MAPT* gene is a risk factor for a variety of neurodegenerative diseases, including AD. Studies have shown that overexpression of *PSEN1* can reduce the activity of the *MAPT* promoter, leading to an increase in methylation of the endogenous *MAPT* promoter, thus causing AD (Coupland et al., 2015). Vitamin B12 (vitB12) and S-adenosylmethionine (SAM), the principal methyl donors, decrease with age in patients with AD (Miner et al., 1997; Beyer et al., 2004). Homocysteine (HCY) is remethylated to methionine, which is, in turn, transformed to SAM. SAM can give the methyl group to DNA (among other substrates) through the action of DNA methyltransferase (DNMT) enzymes family with formation of S-adenosylhomocysteine (SAH). SAH, an inhibitor of DNMTs, is then transformed to HCY. Essential cofactors of HCY metabolism are folate, vitB12 and vitB6. And high HCY and low vitB (folate, vitB12 and vitB6) levels are positively associated with LOAD (GilletteGuyonnet et al., 2007; Luchsinger et al., 2007). Studies have shown that in human neuroblastoma cells and the specific brain regions of AD mouse model, such as the frontal cortex and hippocampus, showing about 3.5- and 1.3-fold increase, respectively, in PSEN1 expression in vitB deficient conditions, whereas SAM is able to restore PSEN1 normal expression (Fuso et al., 2007). PSEN1 5′-flanking region has a site-specific (only few CpG moieties) methylation pattern that could change in response to metabolic stimuli, vitB deficiency (resulting in hyperhomocysteinemia in mice) causes PSEN1 overexpression through DNA demethylation which can be prevented by SAM (Fuso et al., 2011). Further study found that in the above-mentioned cells and mouse brain, the level of overall cytosine methylation is very low in control and almost unaltered in vitB deficiency conditions. However, it is worthwhile to underline that overall CpG methylation is high and significantly decrease when vitB deficiency (Fuso et al., 2011). It means that vitB deficiency mainly affects CpG methylation, through site-specific regulation of cytosine methylation regulating PSEN1 expression (Dong et al., 2000; Lucarelli et al., 2001). Interestingly, after SAM treatment, not only CpG methylation but also non-CpG methylation increased significantly (Fuso et al., 2011). It is not clearly, at present, whether an increase in non-CpG methylation would affect the expression and function of *PSEN1* (Grandjean et al., 2007; Luchsinger et al., 2007). Significance and functions of non-CpG methylation in mammals is still a new and not well-known research field (Grandjean et al., 2007). In addition, during the early stage of AD, the formation of Aβ and tau tangles can demethylate beta-site amyloid precursor protein cleaving enzyme 1 (bace-1) DNA in the brain, while administration of SAM can eliminate this hypomethylation and restore cognitive function (Do Carmo et al., 2016). Other studies have shown that DNA methylation often interacts with multicomb-inhibitory complex 2 (PRC2), resulting in reduced silencing of neurodegenerative genes involved in PRC2 and thus affecting neural development (Zhang et al., 2021).

Mild cognitive impairment (MCI) is a heterogeneous disease, and the prevalence varies greatly according to the environment, follow-up years, medical clinic and other factors (Mitchell and Shiri-Feshki, 2008; Arevalo-Rodriguez et al., 2021). Patients do not show clinically significant memory impairment and may be classified as amnestic or non-amnestic (Petersen, 2004). However, with the passage of time, some patients with MCI may gradually develop progressive cognitive decline and changes in personality and behavior, eventually evolving into dementia, particularly AD (Mitchell and Shiri-Feshki, 2008; Brayne et al., 2011). At the present time, no “cure” for AD is known, but early treatments can slow the cognitive and functional decline and reduce the associated behavioral and psychiatric symptoms of AD (Clare et al., 2003; Birks, 2006). In addition, accurate early identification of AD (MCI) may increase opportunities for the use of newly developed interventions designed to delay or prevent progression to more debilitating stages of the disease. Studies have shown that in patients with MCI, DNA methylation levels of CpG_ 19 of NUDT15 and CpG_11 of TXNRD1, which are redox-related genes, had significantly negative correlations with folate and positive correlations with Hcy, and the interactions of folate and Hcy with DNA methylation could influence cognitive performance (An et al., 2019). Peptidase M20-domain-containing protein 1 (PM20D1), a biosynthetic enzyme for a class of N-lipidated amino acids *in vivo*, is associated with the development of AD. Researchers have shown that the initial promoter hypomethylation of PM20D1 during MCI and early-stage AD is reversed to eventual promoter hypermethylation in late-stage AD, which helps to complete a fuller picture of methylation dynamics (Wang et al., 2020).

In recent years, many biomarkers of aging based on DNA methylation have been developed, such as the multitissue DNA methylation-based (DNAM) age estimator, also known as Horvath’s clock, phenotypes of age estimator, and single-tissue age estimator (Hannum’s clock) (Horvath and Raj, 2018; Levine et al., 2018; Lu et al., 2019). It has been reported that the DNAM epigenetic clock in the AD cortex is associated with AD neuropathological phenomena such as diffuse plaques, neuritic plaques and amyloid accumulation and is related to the decline in the overall cognitive and memory function of AD individuals (Levine et al., 2015); the higher the cortical DNAM age is, the lower the proportion of neuronal cells (Shireby et al., 2020). Although it is not clear what functional aspects of aging can be detected by these markers, examining the relationship between these DNA methylation-based biomarkers of aging and age-related performance indicators may be an approach to assessing healthy aging, of which behavioral and cognitive functions are important components (Lara et al., 2015; Beard et al., 2016).

At present, research on DNA methylation in AD has been relatively mature, involving blood, cerebrospinal fluid and different regions of the brain (Sanchez-Mut et al., 2013; Dabin et al., 2020; Yang et al., 2021). For example, an increase in DNA methylation at 208 CpG sites in the homeobox gene (Hox) cluster is significantly associated with AD neuropathology in the prefrontal cortex and superior temporal gyrus (Smith et al., 2018). In the hippocampus, entorhinal cortex, dorsolateral prefrontal cortex and cerebellum, 130 differentially expressed genes were screened, and their expression was related to DNA methylation (Semick et al., 2019). Methylation of genes, including ABCA7, BIN1, SORL1, and SLC24A4, was found to be significantly associated with Aβ load and tau entanglement in the dorsolateral prefrontal cortex (Yu et al., 2015). Although no treatment based on DNA methylation has been developed, there is no doubt that DNA methylation is a potential therapeutic target for AD.

## Histone Modification

The nuclear duplexed DNA of eukaryotic cells wrapped around histones and organized into chromatin (Margueron and Reinberg, 2010) (Figure 1). Both histones and DNA can affect chromosome structure through covalent modification and then regulate gene expression. There is sufficient evidence about the cytotoxicity of histones (Xu et al., 2009; De Meyer et al., 2012; Guo and Cui, 2018; Cheng et al., 2019), and some studies have shown that extracellular histones can mediate apoptosis, tissue injury and death in mouse models by triggering the TLR2/TLR4 signaling pathway (Xu et al., 2011). In addition, it has been reported that nuclear-like proteins similar to eukaryotic histones can prevent promoters from binding to transcription factors and inhibit gene expression at the genomic level (Dorman, 2004). These results may suggest the side effects of histones on gene expression to some extent.

Histone posttranslational modification (PTM), also known as epigenetic markers, mainly includes methylation, acetylation, phosphorylation, ubiquitination, glycosylation, and ADP ribosylation, which can affect gene expression by changing chromatin structure (Ramazi et al., 2020). Studies have shown that histone markers are significantly correlated with the pathological features of AD, such as abnormal tau phosphorylation and Aβ protein plaques (Narayan et al., 2015).

### Histone Methylation in Alzheimer’s Disease

Histone methylation (the addition of methyl groups to the N-terminal of lysine or arginine under the action of histone methyltransferase) can change the structure of chromatin (Figure 1), resulting in the requisite involvement of chromatin in DNA-based processes including transcription, replication and DNA repair (Kouzarides, 2007). Lysine can be monomethylated, dimethylated and trimethylated, and arginine can be monomethylated and dimethylated (Shi et al., 2006). Lysine methylation at the fourth position of histone H3 (H3K4) is one of the most studied histone methylations and is related to gene expression activation. In addition, H3K4 methylation is associated with synaptic transmission, shaft bursts, and nerve development, all of which affect the development of AD (Cheung et al., 2010). Histone H3K4 trimethylation (H3K4me3) may promote the expression of memory-related genes and proteins such as *ZIF268* and brain-derived neurotrophic factor (BDNF) (Gupta et al., 2010). Among them, *ZIF268* plays an important role in the maintenance of long-term potentiation (LTP) in the hippocampus, and knocking out *ZIF268* in the hippocampus during object recognition memory (ORM) reintegration deletes active recognition memory traces (Gonzalez et al., 2019). The deletion of the H3K4 methyltransferase KMT2B can significantly reduce the levels of H3K4me2 and H3K4me3, leading to the differential expression of some genes, such as *Egr1*, *c-Fos*, and *GluR1*, in the hippocampus. Among them, downregulation of the learning-dependent synaptic plasticity genes *Egr1*, *CREB* and *GluR1* resulted in impaired memory function in mice (Kerimoglu et al., 2013). Experiments have shown that APP-mediated reduction of histone H4 acetylation can also downregulate the transcription of *Egr1*, *c-Fo*s, and BDNF (Hendrickx et al., 2014), thereby affecting synaptic formation and memory function. RAN, a key nuclear and cytoplasmic transport molecule that is significantly decreased in neurons of patients with AD, was only weakly expressed in the nucleus. Some studies have confirmed that a defect of RAN may cause H3K4me3 to accumulate abnormally in the cytoplasm due to its inability to enter the nucleus and then it co-distributes with early tau markers PG5 and MC1. However, it appears earlier than these markers, suggesting that H3K4me3 cytoplasmic accumulation is one of the earliest manifestations of AD cell pathology (Mastroeni et al., 2013, 2015).

Injection of rn-1 into AD model mice to inhibit the demethylation of histone K4 and K9 mediated by the LSD1 enzyme (Jarome and Lubin, 2013) can prevent the memory recognition of new objects in mice (Neelamegam et al., 2012), and LSD1 can combine with deacetylase (KDAC2) to form an inhibitory complex, which seriously affects the normal gene expression and cognitive function of AD mice (Gräff et al., 2012). In addition, the level of SAM is significantly decreased in the AD brain (Morrison et al., 1996), suggesting there may be a decrease in histone methylation in the AD brain.

A number of experimental results have confirmed that histone methylation interferes with AD mainly by affecting the expression of genes and proteins related to learning and memory, synaptic transmission and neuronal growth.

### Histone Acetylation in Alzheimer’s Disease

Histone acetylation is the addition of an acetyl group to the N-terminal lysine residues of histones by histone acetyltransferases (HATs), which leads to a more open chromatin structure (Figure 1). Compared with methylation, histone acetylation has a tendency to promote gene expression (Strahl and Allis, 2000; Peixoto and Abel, 2013; Schneider et al., 2013). In particular, histone acetylation in the central nervous system plays a key role in regulating the expression of genes related to learning and memory (Kosik et al., 2012).

Histone acetylation is catalyzed by five histone lysine acetyltransferase families [KAT2A/GCN5, KAT2B/P300/CBP-associated factor (PCAF), KAT6–8, and CREBBP/cAMP reaction element binding protein (CBP), EP300] (Huynh and Casaccia, 2013). Among them, CBP and P300 play a neuroprotective role in the development of AD, and their abnormal expression leads to neuronal apoptosis and neurodegenerative disease through activation of caspase-6 (Rouaux et al., 2003, 2004; Valor et al., 2013). P300 inhibitors can inhibit the expression of *PSEN1* and *bace1* by reducing H3 acetylation in the promoter region (Lu et al., 2014; Kizuka et al., 2015). Some studies have shown that the acetylation of H2B and H4 in the hippocampus of rats can enhance the expression of memory-related genes, thereby enhancing their spatial memory ability (Li Y. et al., 2021). Moreover, the acetyltransferase Tip60 induces gene transcription by forming a polymer in the cytoplasm with a ligand of APP and an intracellular subdomain of APP, which increases the acetylation of Tip60-dependent histone H3K14 and H4K5 (Smith et al., 2021). This acetylation leads to a decrease in the expression of cytoskeleton-associated proteins and damages the stability of microtubules, thus affecting the NFTs of AD (Barbato et al., 2005; Müller et al., 2013; Sun et al., 2015). Experiments have shown that increasing the Tip60 level in *Drosophila melanogaster* can effectively prevent cognitive deterioration and amyloid accumulation (Xu et al., 2014), suggesting that Tip is neuroprotective. In contrast, it was found that the levels of histone deacetylases (HDACs), such as HDAC1, HDAC3, HDAC4, and HDAC6, in patients with MCI and AD were significantly higher than those in the control group (Mahady et al., 2018). The removal of histone acetyl from nucleosomes by neuron-specific overexpression of HDAC2 can promote chromatin densification and reduce the transcription of corresponding genes, thus reducing synaptic sensitivity, synaptic number, synaptic plasticity and memory function (Guan et al., 2009; Lillico et al., 2016). For example, in wild-type mice and rats, synaptic plasticity and memory formation are promoted after treatment with HDAC inhibitors (Fischer et al., 2007; Vecsey et al., 2007). It was found that the acetylation level of histone H4 is significantly decreased in the frontal cortex and hippocampus of AD transgenic mice and primary neurons cultured from AD transgenic mice, while the HDAC inhibitor 4-PBA can increase the gene transcription of many genes and reverse the spatial learning and memory impairment of AD mouse models (Ricobaraza et al., 2009).

The above studies have shown that HAT and HDAC inhibitors can increase the level of histone acetylation, enhance the expression of memory-related genes, prevent cognitive degeneration and Aβ protein deposition, affect abnormal tau phosphorylation, and reduce NFT formation, thus delaying the progression of AD, while deacetylase has the opposite effect.

### Histone Phosphorylation in Alzheimer’s Disease

Phosphorylation refers to the process of adding phosphate groups to intermediate metabolites or proteins under the action of phosphotransferase (Figure 1). Protein phosphorylation usually occurs on serine or threonine residues (Smith, 1998).

H2AX is a member of the H2A histone family and a component of the nucleosome structure. It has been found that when DNA damage occurs in astrocytes, the serine at position 139 (S139) of H2AX is rapidly phosphorylated to produce γH2AX, while the level of γH2AX in astrocytes in AD susceptible regions (hippocampus and cerebral cortex) increases significantly, suggesting that there is a close relationship between H2AX phosphorylation in astrocytes and AD (Myung et al., 2008). In addition, in neuroblastoma with overexpression of APP, it was found that the S47 phosphorylation level of H4 was 1.89 times higher than that of the control group, and significant phosphorylation of H4 was also found in the brains of patients with mild cognitive impairment, suggesting that this histone phosphorylation may play a role in promoting the pathological development of AD (Chaput et al., 2016). H3 is mainly distributed in the hippocampal CA-1 region and hypothalamus. Studies have shown that phosphorylated H3 is increased in hippocampal neurons in patients with AD, and the activated phosphorylated H3 is mainly confined to the cytoplasm of neurons, which can lead to neuronal mitotic disorders, neurodegeneration and apraxia (Ogawa et al., 2003).

### Histone Ubiquitination in Alzheimer’s Disease

Ubiquitin is a small protein that is highly conserved in eukaryotes, and ubiquitination means adding one or more ubiquitin molecules to the substrate protein for reversible PTM. These modifications cause proteins to undergo proteasome-dependent degradation or change their location or activity in various cellular processes (Martín-Villanueva et al., 2021). Ubiquitination plays a decisive role in clearing toxic metabolites accumulated in the brain through the ubiquitin proteasome system (Kumar et al., 2020). It has been confirmed that E3 ubiquitin ligase is related to the production of Aβ (Benvegnù et al., 2017) and that ubiquitin factor E4B can regulate the ubiquitination of APP, which in turn affects AD (Monica et al., 2020).

Some studies have shown that the ubiquitination of H2B K120 in the brains of patients with AD is 91% higher than that of the control group (Anderson and Turko, 2015) (Figure 1). *Bmi1* is one of the components of PRC1, and the *Bmi1*/Ring1 protein complex can activate E3-momo-ubiquitin ligase on H2A K119 and ubiquitin H2A K119 to maintain transcriptional inhibition of developmental genes (Buchwald et al., 2006; Li et al., 2006) (Figure 1). In the AD brain, *Bmi1* is silenced, and H2A ubiquitination is significantly decreased, resulting in Aβ protein deposition, p-tau accumulation and neurodegeneration (Anthony et al., 2018). In addition, learning-induced monoubiquitination of histone H2B (H2Bubi) is required for increases in the transcriptionally active H3K4me3 mark at learning-related genes in the hippocampus, and the loss of H2Bubi prevents learning-induced increases in H3K4me3, gene transcription, synaptic plasticity, and memory formation (Jarome et al., 2021).

The study of histone ubiquitination in the brain and learning is still in the exploratory stage, but the limited results suggest that there are relationships between histone ubiquitination and AD.

## Non-Coding RNA in Alzheimer’s Disease

Non-coding RNAs (ncRNAs) are a vast and diverse family of non-protein-coding transcripts that modulate cell function by controlling gene expression programs through many different mechanisms, including microRNAs (miRNAs), long non-coding RNAs (lncRNAs), and circular RNAs (circRNAs) (Morris and Mattick, 2014). Among these, lncRNAs are defined as RNAs that have a transcript length exceeding 200 nucleotides and will not be translated into proteins (Dahariya et al., 2019), and a large repertoire of these have been certified to regulate cellular processes, such as chromosome and genome modification, transcription activation, and interference, and nuclear transport, thus driving more researchers to explore how lncRNAs influence human biology (Chen et al., 2021). The mechanisms by which lncRNAs regulate gene expression are rather complicated, studies have found that they can bind to DNA directly or transcription factors, target mRNAs, miRNAs, or proteins and modulate their activities and stability, and can also interfere with chromatin complexes to repress or activate gene expression in an epigenetic fashion (Marchese et al., 2017; Salviano-Silva et al., 2018; Fernandes et al., 2019).

More recently, a growing body of evidence has begun to emphasize lncRNAs modulation in other diverse physiological and pathological processes. Among these, neurological is an area of particular interest, especially in AD. Several lncRNAs, such as BACE1-AS, 51A, BC200, and NDM29, have been found to be aberrantly expressed in AD compared with healthy controls and were involved in AD pathogenesis by the low-throughput experiments (Faghihi et al., 2008; Lin et al., 2008; Massone et al., 2012; Ciarlo et al., 2013). In addition, it has been reported that there is a region-specific and age-dependent expression of lncRNAs in AD and control groups, such as a significant increase in brain precentral gyrus and superior frontal gyrus, and becomes more significant with age (Zhou et al., 2019). These findings demonstrated that the regulation of lncRNAs networks exerts unneglectable influence on the pathology of AD and that lncRNAs may shed new light on the unclear etiology of AD and the current unsatisfactory drug therapy. microRNAs, as the most studied lncRNAS in AD, are also the focus of our attention.

microRNAs (miRNAs) are posttranscriptional gene silencing factors that are a class of 22 nt long non-coding regulatory RNA molecules. By binding to the 3′ untranslated region (UTR) of specific gene mRNAs, miRNAs can induce mRNA degradation or inhibit translation, leading to gene silencing. miRNAs have been predicted to regulate up to 90% of human genes (Miranda et al., 2006) and may control every cellular process in all cells and tissues of the human body. Among all known miRNAs, approximately 70% are expressed in the brain (Provost, 2010).

Studies have shown that differentially expressed miRNAs and differentially expressed target genes are found in the parietal and frontal lobes of the brain, where AD is most likely to occur. Then, through further functional analysis and data mining, it was found that the downregulation of miR-26b-5p, miR-26a-5p, miR-107, and miR-103a-3p in the parietal lobe and the upregulation of HA-miR-7, HA-miR-128, HA-miR-29c, HA-miR-136 in the frontal lobe are closely related to AD (Li J. et al., 2021) (Table 1). It has been reported that miRNA-7, miRNA-9-1, miRNA-23a/miRNA-34a, miRNA-125b-1, miRNA-146a, and miRNA-155 are significantly increased in the AD-affected superior temporal lobe neocortex (Pogue and Lukiw, 2018) (Table 1).

**TABLE 1:** Localization and changes of partial miRNA expression in patients with AD and related models.

miRNAs	Locations	Changes in AD	References
miR-26b-5p	Parietal lobe	↓	Li J. et al., 2021
miR-26a-5p			
miR-107			
miR-103a-3p			
miRNA-188-5p	Hippocampus	↓	Lee et al., 2016
miRNA-485	Hippocampus	↑	
miRNA-4723			Zolochevska and Taglialatela, 2020
miRNA-149			
HA-miR-7	Parietal lobe	↑	Li J. et al., 2021
HA-miR-128			
HA-miR-29c			
HA-miR-136			
miRNA-7	Superior temporal lobe neocortex	↑	
miRNA-9-1			
miRNA-23a/miRNA-34a			
miRNA-125b-1			Pogue and Lukiw, 2018
miRNA-146a			
miRNA-155			
miRNA-146b-5p	Blood	↓	Wu et al., 2020
miRNA-15b-5p			
miRNA-483-5p	Plasma	↑	Sabry et al., 2020

The miRNA-485, miRNA-4723, miRNA-149, and miRNA-200 families have also been found to be differentially expressed in AD and control groups, and their dynamic balance is important for the interaction between Aβ and synaptic terminals and may drive synaptic resistance to Aβ toxicity, thus contributing to the maintenance of cognitive ability (Higaki et al., 2018; Zolochevska and Taglialatela, 2020) (Table 1). Moreover, downregulation of miRNA-188-5p leads to synaptic and cognitive dysfunction, which is eliminated by its overexpression (Lee et al., 2016). HSA-miR-219 has been found to promote neurodegeneration through posttranscriptional regulation of the tau protein. Increased expression of miR-15a can inhibit extracellular signal-regulated kinase (ERK) 1/2 and tau protein phosphorylation, thereby improving cognitive dysfunction in mice, alleviating pathological damage in the hippocampus of AD mice, and inhibiting hippocampal neuronal apoptosis (Li X. et al., 2020; Yang et al., 2020) (Shown in Figure 1 and Table 1).

In addition, some types of miRNAs have been shown to be associated with AD susceptibility and are potential blood biomarkers of AD (Yılmaz et al., 2016). For example, the expression levels of miRNA-146b-5p and miRNA-15b-5p, which are related to innate immunity and apoptosis, are downregulated in the blood of AD patients and are significantly positively correlated with brain amyloid, while they are not brain- or AD-specific miRNAs (Wu et al., 2020). The content of miRNA-483-5p is positively correlated with age and the Dementia Rating (DR) scale, and plasma miRNA-483-5p, as a non-invasive biomarker for the early diagnosis of mild cognitive impairment, is significantly increased in patients with AD (Sabry et al., 2020) (Figure 1 and Table 1).

In addition, microglia, the resident immune effector cells of the central nervous system, are indispensable regulators that initiate the inflammatory response, Aβ aggregation, neuronal loss and memory impairment in AD (Hanisch and Kettenmann, 2007; Clayton et al., 2017). Moreover, the formation of inflammatory bodies and the activation of the protease caspase-1 can cause the release of IL-1β and IL-18 (Man et al., 2017). The increase in these inflammatory mediators is significantly related to the severity of AD (Forlenza et al., 2009; King et al., 2018). Many studies have shown that innate immune signals and activation of inflammatory bodies are a defense mechanism in patients with AD, but overactivation can lead to neuroinflammation and brain damage. Balancing the host’s innate immune response has always been considered a potential means for the treatment of AD (Heneka et al., 2014; Venegas et al., 2017).

## Conclusion and Prospects

The pathogenesis of AD is complex and still unknown, and the prevention and treatment of AD is a global problem. As mentioned above, a growing body of evidence suggests that epigenetic factors are involved in the course of AD and that various epigenetic changes closely influence the development of AD. However, additional studies are necessary to determine whether these epigenetic changes are the cause of AD or the result of AD development and the exact role they play in the pathogenesis of AD.

In this review, we summarized the results of many experimental studies and found that in AD. As Figure 1 shown: DNA methylation regulates the expression of AD-related genes under the action of related enzymes, accelerates the pathological process and aggravates the development of AD; (2) decreased histone methylation leads to synaptic transmission, neuronal growth and memory dysfunction; and (3) changes in related enzymes leads to a decrease in the level of histone acetylation, which leads to the inactivation of memory-related genes and abnormal phosphorylation of tau, resulting in cognitive degeneration. HATs and HDACs inhibitors can reverse these changes and prevent AD; (4) the increased phosphorylation of histones can be related to the pathological phenomenon of AD, resulting in memory impairment; (5) ubiquitination changes can cause Aβ deposition and lead to neurodegeneration; and (6) some miRNAs can lead to synaptic and cognitive dysfunction, and others can inhibit neuronal apoptosis and pathological damage and improve the intelligence of AD mice. Moreover, due to the differential expression of miRNAs in AD, a number of miRNAs have been proposed as blood markers for the early diagnosis of AD. Epigenetic mechanisms may regulate the expression of related genes in the early stage of the disease, and thus, changing the factors related to the development of the disease in patients with AD could be used for the prevention and treatment of AD.

These changes suggest potential therapeutic research directions for AD. Shown by Figure 1, DNA methylation modulators (such as SAM) can reduce the hypomethylation of the AD-related gene *bace-1* and inhibit the pathological aggregation of Aβ, improving cognitive function (Klose and Bird, 2006). HDAC inhibitors have been shown to reduce the amount of phosphorylated tau related to learning and memory in the brain and downregulate the aggregation of tau associated with neuronal apoptosis to improve cognitive dysfunction in mice (Fan et al., 2018). Recent studies have found that a synthetic bs-5-YHEDA peptide (Zou et al., 2019) decreased the methylation of H3 histone levels in the brains of senile mice but enhanced acetylation. Furthermore, by phosphorylating the transcription factor p-ETS1, the bs-5-YHEDA peptide reversed the transcription of SLC40A1 and upregulated ferriportin in the brains of senile mice, thus enhancing the excretion of iron accumulated in the aging brain and consequently protecting neurons and alleviating symptoms such as AD (Figure 1). That is, intervening in epigenetics may block the progression of AD or improve the condition of the patients. Epigenetic interference is a potential therapeutic for AD, and developing relevant drugs is promising for the treatment of AD. In addition, the results have shown that most of the epigenetic phenomena in AD are related to the pathology of AD, and some epigenetic changes may appear before the pathological changes, suggesting that these epigenetic changes may provide a diagnostic tool for AD and that targeting these changes could be a way to prevent and treat AD.

## Author Contributions

XG, ZZ, and JT: conceptualization, methodology, writing, reviewing, and editing. QC, HY, YZ, and ZL: analyzing supervision. All authors contributed to the article and approved the submitted version.

## Conflict of Interest

The authors declare that the research was conducted in the absence of any commercial or financial relationships that could be construed as a potential conflict of interest.

## Publisher’s Note

All claims expressed in this article are solely those of the authors and do not necessarily represent those of their affiliated organizations, or those of the publisher, the editors and the reviewers. Any product that may be evaluated in this article, or claim that may be made by its manufacturer, is not guaranteed or endorsed by the publisher.

## Abbreviations

AD: Alzheimer’s disease
ANK1: ankyrin gene
APP: amyloid precursor protein
BACE1: β-site amyloid precursor protein cleaving enzyme
bace-1: β-site amyloid precursor protein lyase 1
BDNF: brain-derived neurotrophic factor
beta4 GalT 7: beta-4-galactosyltransferase 7
CBP/CREBBP: CREB binding protein
CKD-504: HDAC6 inhibitor
CREB: cAMP-response element binding protein
DNAM: DNA methylation-based
DNMT: DNA methyltransferase
Egr1: early growth response protein 1
elF-2 α: eukaryotic initiation factor-2 α
GluR1: glutamate ionotropic receptor AMPA type subunit 1
H1: histone 1
H2A: histone 2 A
H2B: histone 2 B
H3: histone 3
H4: histone 4
HATs: histone acetyltransferases
HDAC: histone deacetylase
KAT6–8: lysine acetyltransferase 68
KDAC2: lysine deacetylase 2
KMT2B: histone-lysine N-ethyltransferase 2B
LSD1: lysine specific demethylase 1
NFTs: neuronal fiber tangles
ORM: object recognition memory
P300: adenoviral E1A binding protein of 300 kDa
PCAF: P300/CBP-associated factor
pgnat5: polypeptide N-acetylgalactosaminyltransferase 5
PRC2: polycomb repressive complex 2
PSEN1: presenilin-1
PTM: posttranslational modification
Tip60: tat interactive protein 60 ku
UTR: untranslated region
ZIF268: zinc finger-containing transcription factor 268.
